# SCF and IL-33 regulate mouse mast cell phenotypic and functional plasticity supporting a pro-inflammatory microenvironment

**DOI:** 10.1038/s41419-023-06139-7

**Published:** 2023-09-20

**Authors:** Rosa Molfetta, Mario Lecce, Nadia D. Milito, Erisa Putro, Giuseppe Pietropaolo, Caterina Marangio, Gianluca Scarno, Marta Moretti, Enrico De Smaele, Tiziana Santini, Giovanni Bernardini, Giuseppe Sciumè, Angela Santoni, Rossella Paolini

**Affiliations:** 1 Department of Molecular Medicine, Laboratory affiliated to Istituto Pasteur Italia - Fondazione Cenci Bolognetti, Sapienza University of Rome, 00161 Rome, Italy; 2 Department of Experimental Medicine, Sapienza University of Rome, Viale Regina Elena 324, 00161 Rome, Italy; 3 Department of Biology and Biotechnologies “Charles Darwin”, Sapienza University of Rome, Rome, Italy; 4 IRCCS Neuromed, Pozzilli, 86077 Isernia Italy; 5 Present Address: Leibniz Institute for Immunotherapy-Division of functional immune cell modulation, Franz-Josef-Strausse, D-93053 Regensburg, Germany; 6 Present Address: Human Oncology and Pathogenesis Program, Memorial Sloan Kettering Cancer Center, New York, NY 10065 USA

**Keywords:** Colorectal cancer, Diseases

## Abstract

Mast cells (MCs) are multifaceted innate immune cells often present in the tumor microenvironment (TME). Several recent findings support their contribution to the transition from chronic inflammation to cancer. However, MC-derived mediators can either favor tumor progression, inducing the spread of the tumor, or exert anti-tumorigenic functions, limiting tumor growth. This apparent controversial role likely depends on the plastic nature of MCs that under different microenvironmental stimuli can rapidly change their phenotype and functions. Thus, the exact effect of unique MC subset(s) during tumor progression is far from being understood. Using a murine model of colitis-associated colorectal cancer, we initially characterized the MC population within the TME and in non-lesional colonic areas, by multicolor flow cytometry and confocal microscopy. Our results demonstrated that tumor-associated MCs harbor a main connective tissue phenotype and release high amounts of Interleukin (IL)-6 and Tumor Necrosis Factor (TNF)-α. This MC phenotype correlates with the presence of high levels of Stem Cell Factor (SCF) and IL-33 inside the tumor. Thus, we investigated the effect of SCF and IL-33 on primary MC cultures and underscored their ability to shape MC phenotype eliciting the production of pro-inflammatory cytokines. Our findings support the conclusion that during colonic transformation a sustained stimulation by SCF and IL-33 promotes the accumulation of a prevalent connective tissue-like MC subset that through the secretion of IL-6 and TNF-α maintains a pro-inflammatory microenvironment.

## Introduction

Mast cells (MCs) are tissue-resident sentinels involved in a large number of physiological and pathological processes [1]. They are recognized as key effector cells of IgE-mediated allergic disorders and they are also important players in regulating innate and adaptive immune responses thanks to their capability to respond to a plethora of different stimuli by releasing pro-inflammatory mediators [1–3]. MCs are also frequently observed in tumors, suggesting their contribution to the transition from persistent inflammation to cancer [4].

MC modulatory roles depend on the expression of several receptors that upon ligand binding can induce the release of stored mediators and the de novo synthesis of lipid metabolites and various chemokines and cytokines. The well-known high affinity receptor for IgE (FcεRI) together with c-Kit (CD117), the receptor for the Stem Cell Factor (SCF), characterize mature MCs that undergo terminal differentiation in target tissues [5, 6]. SCF is the best-described MC growth and differentiation factor as exemplified by the tight connection between activating *kit* mutations and systemic mastocytosis [7]: in the tissue microenvironment SCF orchestrates MC development and ensure the survival of mature MCs [5, 7, 8].

Together with SCF, other soluble mediators can support MC activation and expansion including a member of the interleukin (IL)-1 superfamily, namely IL-33 [9]. Upon danger signals, IL-33 is the most potent alarmin among several stimuli to promote MC activation [10, 11] and is released by many tissue-resident cells, such as epithelial and endothelial cells [12, 13]. Of note, MCs act as critical amplifiers in IL-33-induced inflammation not only for their abundant surface expression of ST2, the IL-33-specific subunit of IL-33 receptor, but also due to their own capability to release IL-33 [10, 11, 14, 15]. IL-33 potentiates IgE-mediated MC responses and cooperate with the responsiveness to SCF by influencing signaling events and secretory MC functions [16–19]; however, the final redout may depend on the environment in which MC differentiates.

In mice, at least two subtypes of fully differentiated MCs can be identified: mucosal MCs (MMCs) that are mainly associated with the lung and gastrointestinal tract epithelia, and connective tissue MCs (CTMCs) mainly found in the intestinal submucosa, peritoneum, and skin [20]. Apart from their anatomic location, these two subsets are distinguished by the expression of specific proteases, with mucosal MCs expressing primarily the chymases mouse Mast Cell Protease (mMCP)1 and mMCP2, and connective tissue MCs expressing mMCP4-to-7 and carboxypeptidase A [20, 21].

However, this traditional classification is simplistic since there are compelling evidence demonstrating that the microenvironment plays a prominent role in shaping MC phenotype and function, thus revealing a high degree of MC plasticity [22, 23]. Moreover, the MC transcriptional profile underscores heterogeneity in gene expression across different connective tissues [24].

Of note, it is not only the tissue site but also the disease status that influences MC heterogeneity.

For instance, in patients with poorly controlled, severe Th2-associated asthma, an altered MC subtype that could play a role in the pathophysiology of this disease has been described [25].

Moreover, during the acute phase of *T. spiralis* infection MMCs expand and through the action of mMCP-1 increase intestinal permeability to facilitate parasite expulsion [26] but in the chronic phase of infection a primary contribution from the CTMC tryptase mMCP-6 has been also reported [27].

MCs have been also identified in different tumors, suggesting their contribution to cancer initiation and progression [28].

In line with this view, several evidence have demonstrated that MCs, and their elaborated proteases, guide the progression from chronic intestinal inflammation to colorectal cancer (CRC) and depending on the type, grade or stage of tumors, the specific role of MCs largely varies being either pro- or anti-tumorigenic [4, 28–30]. However, a comprehensive description of how the intestinal tumor microenvironment (TME) shapes MC phenotypes and effector functions, is currently missing.

Using a mouse model of chemical-induced inflammatory colorectal cancer, herein we report that CRC microenvironment is characterized by high amounts of SCF and IL-33. During the development of cancer under the action of SCF CTMCs accumulate in colonic lesions and, despite a dramatic down-modulation of c-kit, they are still capable of producing high amounts of pro-inflammatory cytokines. Similarly, when IL-33 and SCF were used in combination to stimulate primary MC cultures they can interact to additively provoke the production of pro-inflammatory cytokines.

## Results

### During colon carcinogenesis activated MCs with a connective tissue-like phenotype accumulate in tumor lesions

To determine the frequency of MCs during tumor development, we utilized a conventional inflammation-driven murine colon cancer model based on the intraperitoneal administration of azoxymethane (AOM), which exerts colonotropic carcinogenicity, with the inflammatory agent dextran sodium sulfate (DSS) in drinking water, which causes growth of multiple colon tumors [31].

Acute colitis and colonic adenomas were induced as schematized in Fig. S1A.

Histologic analysis showed the loss of crypt architecture going from colitis to tumor (Fig. S1B). Moreover, Ki67, a marker of cell proliferation and growth, revealed a high grade of epithelial cell proliferation suggestive of active transformation in the tumoral colon tissue of AOM/DSS-treated mice (Fig. S1C).

**Fig. 1:**
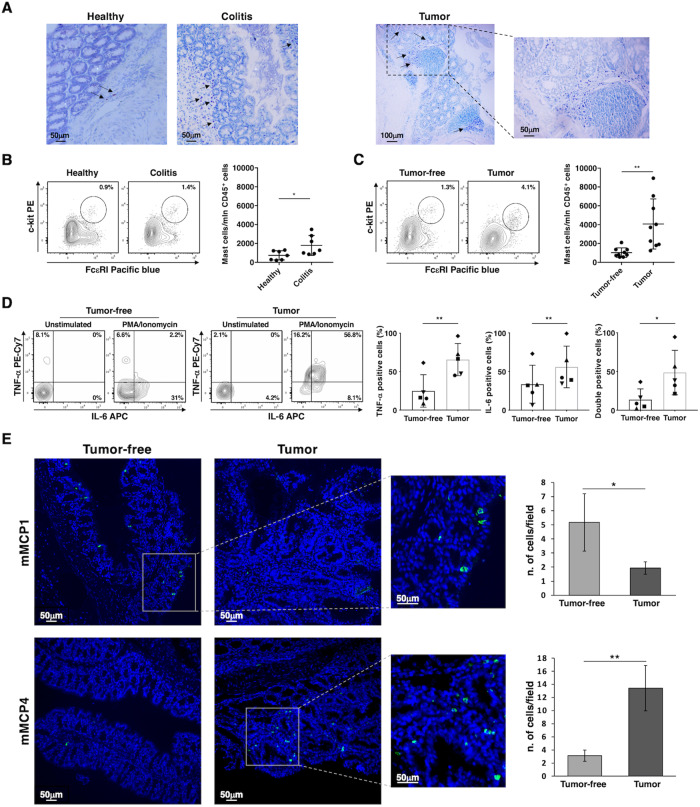
Evaluation of MC frequency and function in DSS and AOM/DSS mouse models. **A** Representative histochemical Toluidine Blue staining for MCs in the colon paraffin-embedded sections of untreated mice (Healthy), in the inflamed colon (Colitis) and inside the AOM/DSS-induced colorectal tumors (Tumor) acquired with 20× objective (Healthy and Colitis) or 10× and 40× (insert) objectives (Tumor). MCs appear purple (arrows). **B** Lamina propria cells were isolated by enzymatic digestion from colon of DSS-induced colitis mice (Colitis) and colon of untreated mice (Healthy). **C** Upon AOM administration and 4 cycles of DSS treatment, colorectal tumors/polyps (Tumor) were dissected and tumor-infiltrating cells were isolated by enzymatic digestion. Cells isolated from the tumor-free colonic counterpart of AOM/DSS treated mice were used as control (Tumor-free). **B**, **C** MCs were identified as c-kit/FcεRI double positive cells gated on CD45^+^/CD19^−^/CD3^−^/NK1.1^−^/CD11b^−^ cells. Representative dot plots (left panels) and number of MCs expressed as mean ± SD of the mice analyzed are shown (right panels). Each vertical bar is representative of 3 independent experiments with at least 2 mice/group. **B** Unpaired Student’s *t* test *p < 0.05. **C** Paired Student’s *t* test **p < 0.01. **D** Cells were stimulated with PMA/Ionomycin for 2 h and the expression of TNF-α and IL-6 was analyzed on c-kit^+^FcεRI^+^ MCs by flow cytometry. A representative dot plot is shown on the left. The right histograms show the percentage of cytokine-positive MCs upon PMA/Ionomycin stimulation. The percentages of IL-6/TNF-α double positive MCs are also shown. Each graph is representative of 2 independent experiments with 5 mice/group. Each symbol represents data obtained from an individual mouse and the same group of mice are indicated with the same symbol. Paired Student’s *t* test **p < 0.01 and *p < 0.05. **E** Immunofluorescence of colon paraffin-embedded sections from AOM/DSS treated mice were stained with anti-MCP1 (upper panels) or anti-MCP4 (lower panels) Abs followed by Alexa Fluor 488 secondary Abs (green). Nuclei were counterstained with DAPI (blue). Images were acquired with 20× objective. The frequencies of MCs positive for mMCP1 and mMCP4 proteases analyzed in 20 fields randomly acquired from three independent experiments with a total of 3 mice/group are shown (right panels) and classified as Tumor-free or Tumor based on normal or aberrant crypt architecture, respectively, as assessed by DAPI signal. Unpaired Student’s *t* test *p < 0.05; **p < 0.01.

To visualize MCs within colon tissue, paraffin-embedded colon sections of untreated and treated mice were initially stained with toluidine blue, a metachromatic marker for MC granules (Fig. 1A). While MCs were only occasionally present in the intestinal tissue of untreated mice, MC number increased both in inflamed colon tissue (DSS treated mice) and even more in the tumor (AOM/DSS treated mice).

MC frequency in the inflamed colon tissue (mice treated with DSS only) and in the tumor/polyps induced upon AOM/DSS treatment, was then evaluated by flow cytometry. MC percentage was also determined in the colon of untreated mice and in colonic tumor-free areas of AOM/DSS-treated mice, as a control. After excluding CD3^+^/CD19^+^/CD11b^+^/NK1.1^+^/dead cells and positive gating for CD45^+^ cells (Fig. S2), we observed an increase of c-kit/FcεRIα double-positive cells in colon of DSS-induced colitis mice with respect to control mice; moreover, we found a more pronounced accumulation in the tumor masses of AOM/DSS mice compared to inflamed colon and tumor-free surrounding tissue (Fig. 1B, C). We then evaluated MC functionality by assessing the presence of two pro-inflammatory cytokines, TNF-α and IL-6, highly produced by MCs. Upon intracellular staining, flow cytometric analysis showed a much higher production of both cytokines in tumor-associated MCs (TAMCs) compared to MC from tumor-free tissue of AOM/DSS treated mice (Fig. 1D).

To identify the subtype of TAMCs, paraffin-embedded colon sections of AOM/DSS treated mice were stained with antibody against mMCP1, which is specifically expressed by MMCs, or with antibody against mMCP4, a chymase observed into the secretory granules of CTMCs, and analyzed by confocal microscopy. The immunofluorescence images of tumor-free and tumor areas double stained for MCP1 and MCP4 proteases clearly show the presence of two distinct MC populations (Fig. S3). Notably, TAMCs mainly express mMCP4 whereas reduced frequencies of mMCP4^+^ MCs and increased number of mMCP1^+^ MCs were found in the adjacent tumor-free tissue (Fig. 1E). Similarly, MCs observed in colon sections from DSS-induced colitis mice were positive for mMCP4 (Fig. S3B).

Thus, MCs showing an activated phenotype and expressing mMCP4 expand in colonic lesions of AOM/DSS mice.

### SCF/c-kit and IL-33/ST2 pathways control MC phenotype and functions

Several evidence demonstrate that microenvironment plays a prominent role in shaping MC plasticity [22–27].

In the intestinal microenvironment, during CRC progression the c-kit ligand SCF and the alarmin IL-33 are both abnormally expressed and are considered biomarkers of poor prognosis for their pro-tumorigenic action [32–36]. Notably, IL-33 elicits a complex between its receptor, consisting of ST2 coupled with IL-1 receptor accessory protein (IL-1RAP), and c-Kit, supporting the finding that IL-33 and SCF can act synergistically to activate MC signaling [16].

**Fig. 2:**
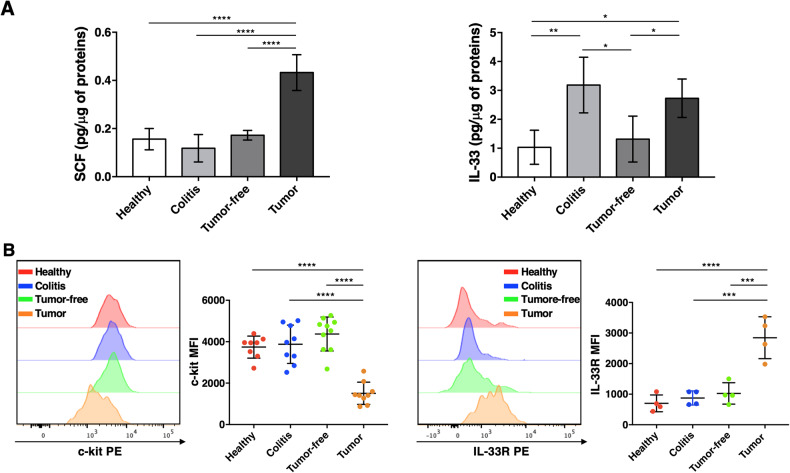
High levels of SCF and IL-33 are present in TME and modulate c-kit and IL-33R expression on MCs. **A** SCF and IL-33 levels were analyzed by ELISA using lysates of colon tissues obtained from untreated mice (Healthy), DSS-treated mice (Colitis) and AOM/DSS treated mice (Tumor-free and Tumor). Cytokine concentrations are expressed as picograms of cytokine per microgram of total proteins in the tissue sample. Means ± SD of three independent experiments are shown. One-way ANOVA *p < 0.05; **p < 0.01; ****p < 0.0001. **B** Expression of c-kit and IL-33R was analyzed on AOM/DSS-induced colorectal tumors/polips (Tumor) compared with the tumor-free colonic counterpart (Tumor-free), colon of DSS-induced colitis mice (Colitis) and colon of untreated mice (Healthy). Left, A representative experiment is shown. Right, Mean fluorescence intensity (MFI) ± SD of the different analyzed mice are shown. Each vertical bar is representative of two (IL-33R) or three (c-kit) independent experiments with at least 2 mice/group. One-way ANOVA ***p < 0.001; ****p < 0.0001.

Thus, we first investigated the presence of SCF and IL-33 on colon tissue lysates obtained from DSS and AOM/DSS treated mice. Accumulation of SCF were observed in tumor lesions compared with tumor-free tissue (colon AOM/DSS) and inflamed colon tissue (colon DSS) (Fig. 2A, left panel), while high levels of IL-33 were detected on both inflamed and tumor lesions (Fig. 2A, right panel). Concurrently, by flow cytometry we evaluated whether the sustained contact with SCF and IL-33 would affect MC surface expression of c-kit and ST2. Down-modulation of c-kit and up-regulation of IL-33R were reproducibly observed on TAMCs compared to surrounding tumor-free tissue and inflamed colon tissue (colon DSS) (Fig. 2B).

To investigate whether and how SCF and IL-33 regulate MC phenotype and functions, we decided to perform in vitro experiments generating primary bone-marrow-derived mast cells (BMMCs), as previously described [37, 38].

**Fig. 3:**
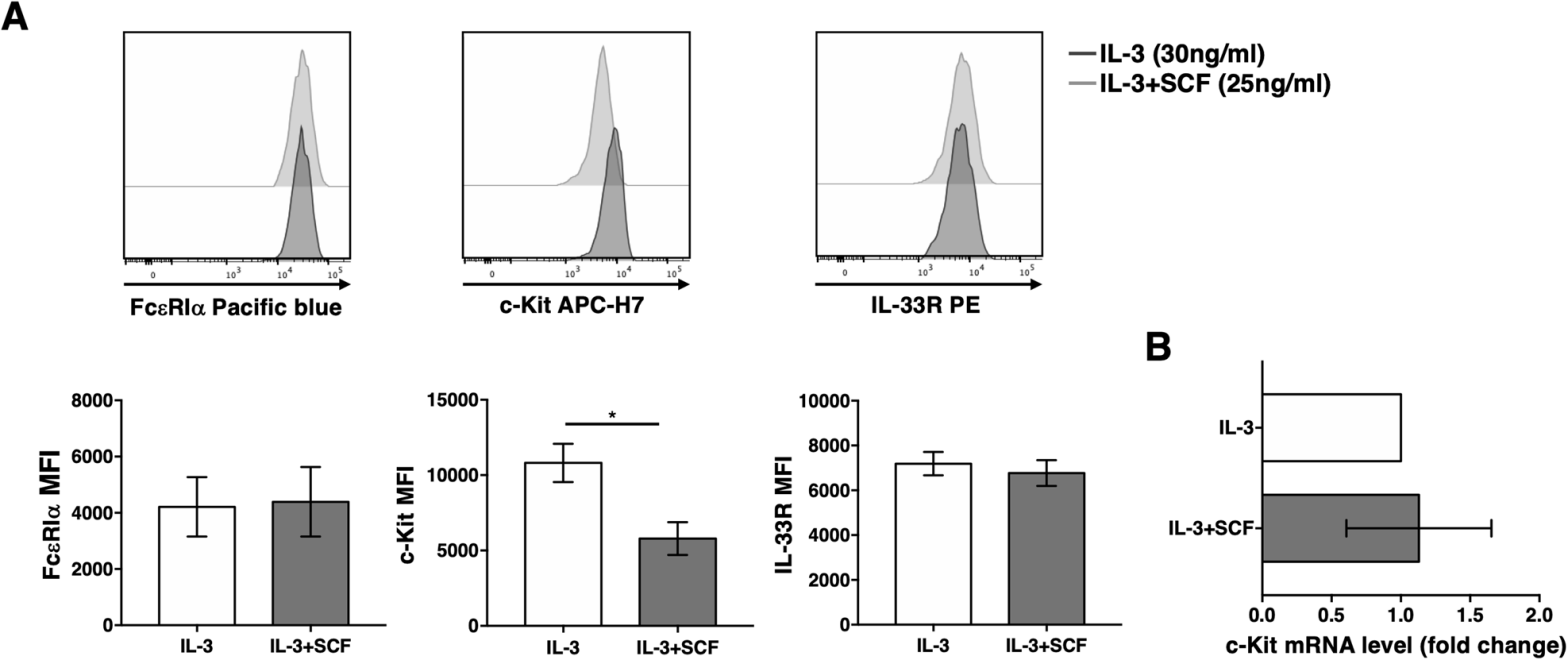
c-Kit is downmodulated on BMMC grown in the presence of SCF. MCs were differentiated in vitro from bone marrow precursors in the presence of IL-3 alone (30 ng/ml) or in combination with SCF (25 ng/ml), as indicated. **A** Upon 4 weeks of culture, the expression of FcεRI, c-kit and IL-33R was analyzed by flow cytometry. Representative histograms are shown in the upper panels, while means +/- SD of three independent experiments are shown in lower panels. Paired Student’s *t* test *p < 0.05. **B** c-Kit expression levels were analyzed by real-time PCR. c-Kit mRNA levels in BMMC differentiated with IL-3 alone was arbitrary set to 1 and results shown as fold change. Means ± SD of three independent experiments are shown.

BM-derived cells were either cultured with IL-3 alone or in combination with SCF, and the generation of pure BMMCs was monitored by flow cytometry evaluating the expression of FcεRI, c-kit, and IL-33R upon 4 weeks of culture. Notably, FcεRI and IL-33R surface expression remained untouched and the expression was comparable in the different cultures, whereas c-kit expression was strongly reduced on BMMCs differentiated in the presence of SCF when compared with MCs cultured with IL-3 alone (Fig. 3A). MCs from both types of 4-week cultures expressed comparable RNA level of c-kit, as revealed by real-time PCR (Fig. 3B).

Interestingly, BMMCs differentiated in IL-3 alone and then subjected to short-term exposure with 25 ng/ml SCF showed a progressive loss of c-kit surface expression accompanied by a slight decrease in total protein levels (Fig. S4), likely due to a partial degradation of engaged receptor complexes (data not shown). On the contrary, MCs differentiated in the presence of IL-3 and SCF and then deprived of SCF overnight partially restored c-kit surface expression (data not shown).

Regardless, c-kit downmodulation induced upon BMMC differentiation in the presence of SCF is accompanied by a defect in response to IgE and multivalent antigen stimulation both in term of degranulation and cytokine production (Fig. S5).

Thus, SCF affects MC phenotype inducing c-kit downmodulation and a concomitant hypofunctional MC state in response to FcεRI engagement.

The presence of SCF during MC differentiation also induced a predominant connective-like phenotype characterized by high transcriptional levels of mMCP4, 5 and 6, while MCs differentiated in the presence of IL-3 showed a significant higher expression of mMCP1 transcripts, as revealed by RT-PCR (Fig. S6).

**Fig. 4:**
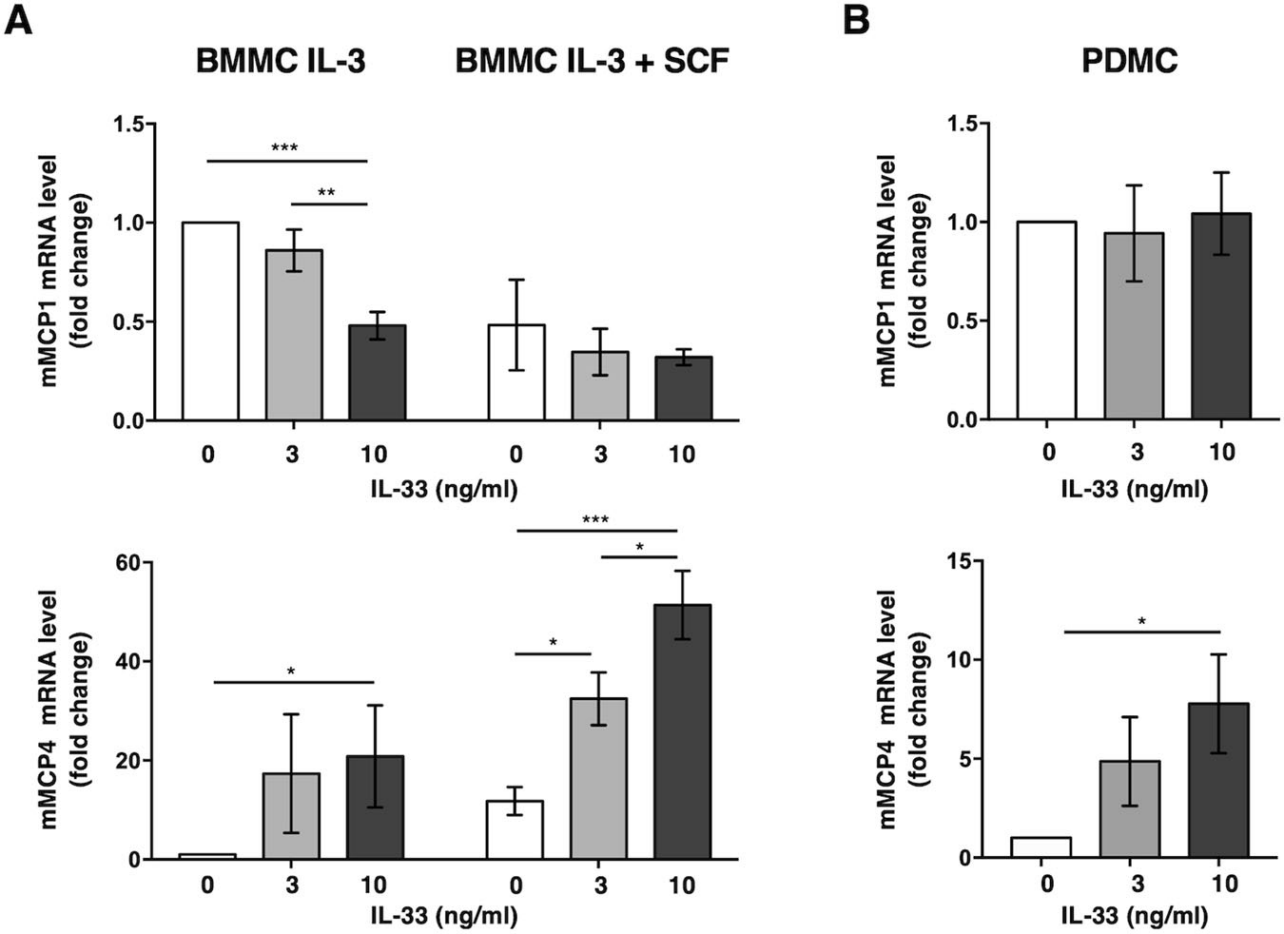
Dynamic changes in protease transcripts along BMMC and PDMC cultures upon IL-33 stimulation. BMMCs (**A**) were differentiated from BM-precursors cultured in the presence of IL-3 alone or in combination with SCF, as indicated in Fig. 4, while PDMC (**B**) were differentiated from isolated peritoneal committed MCs cultured in the presence of IL-3 (30 ng/ml) and SCF (25 ng/ml) for 10 days. BMMCs and PDMC were stimulated with the indicated doses of IL-33 for 48 h and the expression levels of mMCP1 and mMCP4 mRNA transcripts were evaluated by real-time PCR. **A** The amount of mRNA expressed in unstimulated BMMC differentiated with IL-3 alone was arbitrary set to 1. Means ± SD of three independent experiments are shown as fold change. One-way ANOVA *p < 0.05; **p < 0.01; ***p < 0.001. **B** The amount of mRNA expressed in unstimulated PDMC was arbitrary set to 1. Means ± SD of three independent experiments are shown as fold change. One-way ANOVA *p < 0.05.

To resemble the TME cytokine milieu, we further evaluated whether and how stimulation with IL-33 affects BMMC phenotype and functions. Interestingly, IL-33 stimulation for 48 h was accompanied by dynamic changes in MC-protease expression without a significant alteration of IL-33R surface expression (data not shown). In particular, mMCP1 levels showed a significant decrease only on BMMCs cultured in the presence of IL-3 alone while a selective fold increase of the transcriptional mMCP4 levels was observed on both BMMC cultures (Fig. 4A), being higher in MCs differentiated in the presence of SCF.

Since BMMCs may not reflect the phenotype of the tissue-derived corresponding MCs, an in vitro cell model that closely resembles intestinal MCs was also established. To this purpose, we have generated primary cultures from isolated peritoneal committed MCs grown in the presence of IL-3 and SCF for 10 days. Consistent with our previous finding obtained on BMMCs, stimulation with IL-33 was able to selectively increase mMCP4 on peritoneal-derived MCs (PDMCs), leaving almost untouched the transcriptional levels of mMCP1 (Fig. 4B). Although less pronounced, an increase in mMCP5 and mMCP6 expression levels were also seen on both BMMC and PDMC cultures (data not shown).

Collectively, these results demonstrate the capability of IL-33 to induce a connective tissue-like MC phenotype and suggest a combined action of SCF and IL-33 in shaping MC plasticity in vivo.

### SCF and IL-33 mediate unique and convergent signals responsible for IL-6 and TNF-α release on primary murine mast cells

**Fig. 5:**
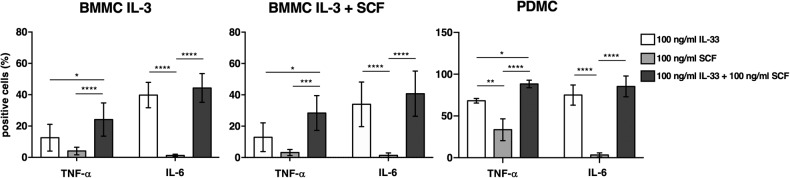
SCF and IL-33 stimulate cytokine production on primary MC cultures. BMMC differentiated in IL-3 + SCF and PDMC primary cultures were starved overnight from SCF and then stimulated with 100 ng/ml of IL-33 or SCF alone or in combination for 6 h in the presence of Brefeldin A. TNF-α and IL-6 expressions were analyzed by flow cytometry on permeabilized cells. Means ± SD of three independent experiments are shown as percentage of cells positive for the indicated cytokines. One-way ANOVA *p < 0.05; **p < 0.01; ***p < 0.001; ****p < 0.0001.

Next, we evaluated the capability of SCF and IL-33 to stimulate cytokine production in MC primary cultures (Fig. 5).

Those soluble factors can provoke a superimposable production of cytokines from both BMMC cultures, regardless the hypofunctional state in response to FcεRI engagement previously observed on IL-3/SCF differentiated BMMCs. Interestingly, PDMCs were able to produce higher amounts of cytokines compared to BMMC cultures in response to both stimuli used alone or in combination. In particular, they responded very well to IL-33 stimulation alone producing high amount of IL-6. Notably, we found that the combined treatment with SCF and IL-33 increased cytokine production over the individual stimuli in all 3 types of primary MC cultures, and that the most accentuated additive effect was observed for TNF-α.

Upon stimulation with SCF in combination with IL-33, different signaling routes can be triggered depending on MC subsets [16, 19, 39, 40].

**Fig. 6:**
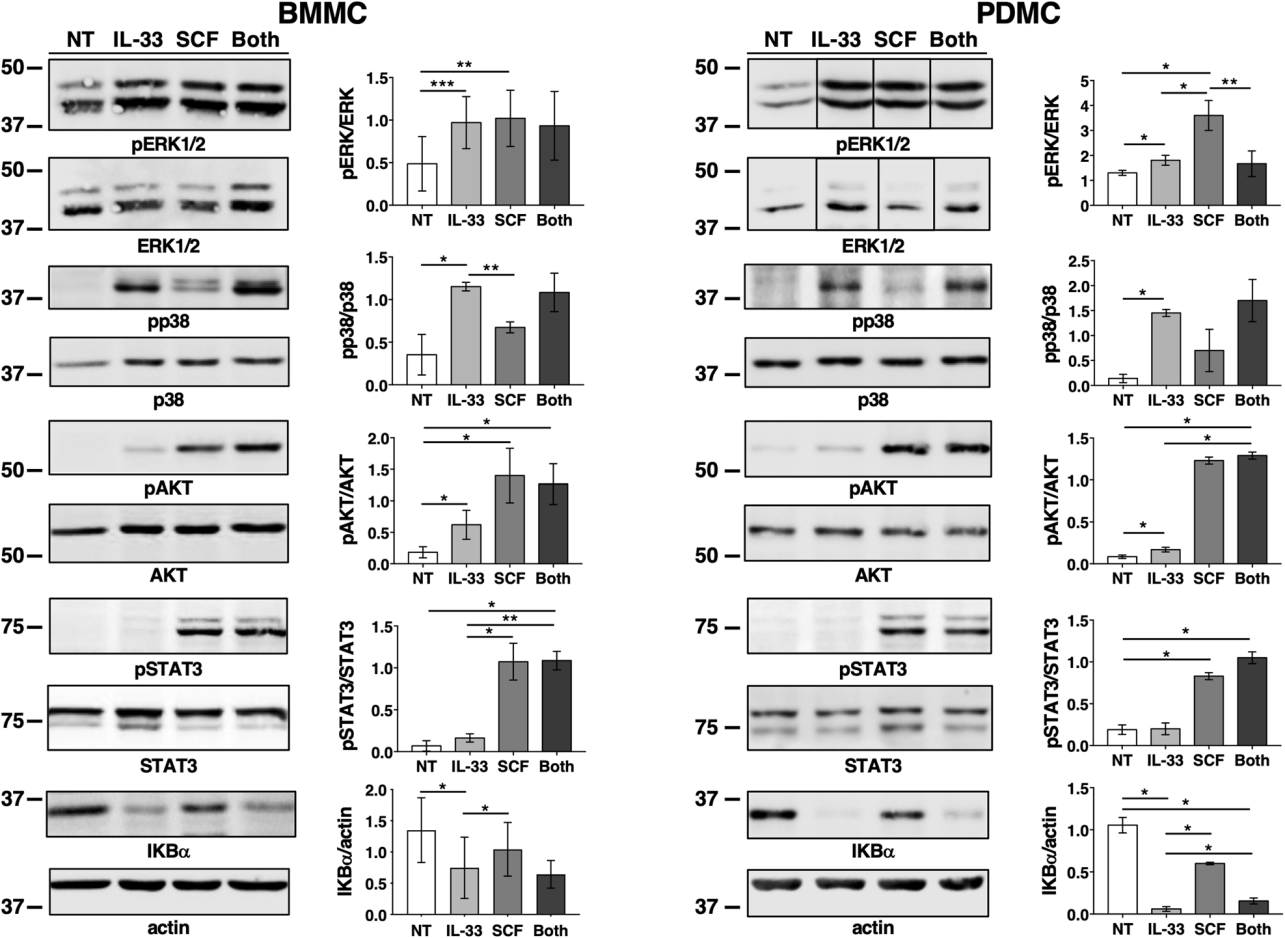
SCF and IL-33 mediate unique and convergent signaling pathways. BMMC and PDMC both differentiated in IL-3 (30 ng/ml) and SCF (25 ng/ml) were starved overnight from SCF prior stimulation with 100 ng/ml of IL-33 and/or SCF for 15 min. Cells were lysed and equal amounts of total proteins were separated on gels run in parallel (SDS-PAGE). Levels of phosphorylated and total proteins were analyzed by western blot, as indicated. One representative result is shown. The pERK and ERK (PDMC) immunoblots are from the same gels but lanes were non-continuous. Densitometric analysis of phosphorylated proteins was performed by FIJI software and normalized with the total protein amount. Quantitative changes in protein phosphorylation upon different stimuli of three or five (for IkBα) different independent experiments are shown in the graphs.

We found that on both MC primary cultures, SCF and IL-33 triggered a robust and comparable phosphorylation of ERK1/2 (Fig. 6). Akt phosphorylation was more efficiently induced by SCF while p38 activation was more evident upon IL-33 stimulation. Notably, SCF selectively induced pSTAT3 whereas IL-33 promoted a significant IkB-α degradation, necessary for NF-kB activation, likely explaining its ability to stimulate high level of IL-6 production even in the absence of SCF (see Fig. 5).

All together these results demonstrate that IL-33 and SCF could activate selective biochemical pathways on primary MC cultures and suggest that their combined action is necessary to provoke a huge production of pro-inflammatory cytokines.

### Connective tissue-like MCs accumulate in tumor lesions in the presence of SCF and are the main cytokine producer

Besides the combined in vitro action of IL-33 and SCF in shaping MC phenotype and functions, a previous finding supports the ability of SCF to favor MC infiltration and activation in the tumor microenvironment [41]. However, no data are available on SCF-dependent recruitment of a selective MC subset.

**Fig. 7:**
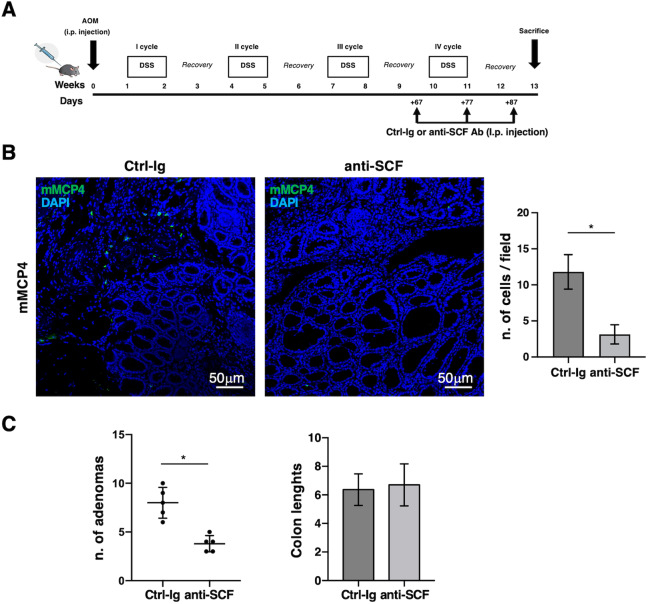
SCF controls connective tissue-like MC accumulation in tumor lesions. **A** Schematic representation of anti-SCF neutralizing Ab administration in vivo. AOM/DSS-treated mice were i.p. injected three times with anti-SCF or control Ab (100 μg/mouse) starting from the fourth DSS cycle. Treated and control mice were sacrificed at 13 weeks from AOM administration. **B** Colon paraffin-embedded sections from AOM/DSS mice treated with Ctrl-Ig or anti-SCF neutralizing antibodies as described in (**A**) were stained with anti-MCP4 Ab followed by Alexa Fluor 488 secondary Abs (green). Nuclei were counterstained with DAPI (blue) and images were acquired with a Zeiss LSM980 confocal microscopy using a 20× objective. The frequencies of MCs positive for mMCP4 protease were analyzed in 20 fields randomly acquired from tumor lesions and shown as mean ± SD cells/field. Paired Student’s *t* test: *p < 0.05. Number of adenomas and colon lengths are shown in (**C**). Paired Student’s *t* test: *p < 0.05. Graphs are representative of two independent experiments with a total of 5 mice/group.

To investigate whether SCF neutralization reduces the accumulation/expansion of MCP4^+^ MC subset, mice were treated (i.p.) with isotype control IgGs (Ctrl-Ig) or SCF-neutralizing antibody before the beginning of the last DSS cycle, as schematically depicted in Fig. 7A.

Compared to untreated mice, SCF neutralization induced a selective decrease of the connective tissue-like MC subset (Fig. 7B) leaving unchanged MCP1^+^ MC subset (data not shown). The reduced number of infiltrating MCP4^+^ MCs was accompanied by inhibition of tumor burden in the absence of any significant difference in colon length (Fig. 7C).

**Fig. 8:**
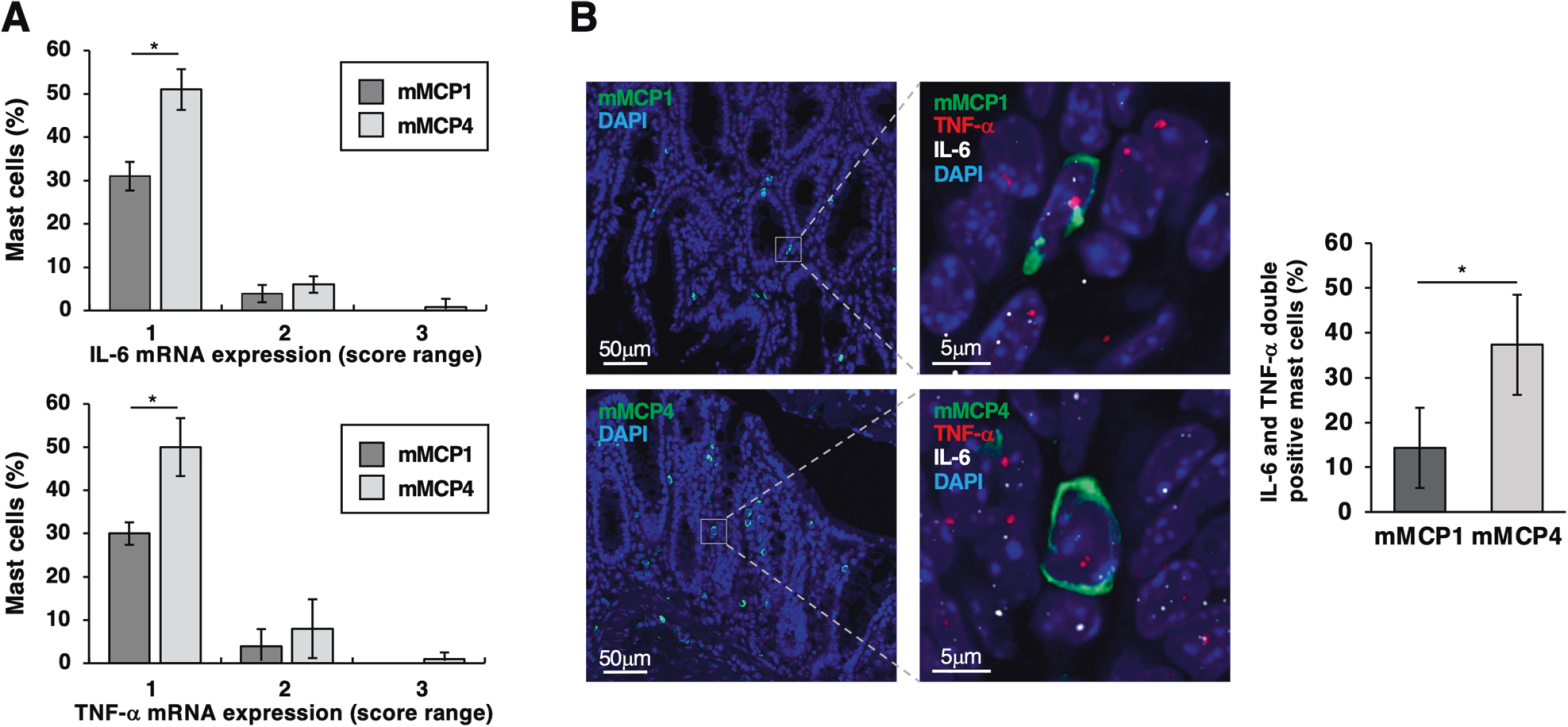
Tumor infiltrating MCP4+ MCs are the main cytokine producing subset. **A**, **B** RNA-Scope assay for cytokines combined with immunofluorescence for MC proteases highlights cytokine production by mucosal and connective MC subsets. The two MC subsets were immunostained for mMCP1 or mMCP4, respectively, while IL-6 and TNF-α mRNA expression was quantified by RNA-scope. **A** Percentage of the two MC subsets (mMCP1 and mMCP4) negative or positive for the indicated cytokines was calculated on the basis of the following four-grade scoring system: score 0, no staining or 1 dot/cell; score 1, 2–4 dots/cell; score 2, 5–10 dots/cell; score 3, > 10 dots/cell. Means ± SD of three independent experiments (30 cells/each exp) are shown. Paired Student’s *t* test: *p < 0.05; **p < 0.01. **B** Low magnification images (20×) show MCs positive for mMCP1 or mMCP4 proteases (green) in transformed colon paraffin-embedded sections of AOM/DSS treated mice. High magnification images (63x) show mMCP1 or mMCP4 MCs (green) double positive for IL-6 (white dots) and TNF-α (red dots) mRNA expression. Nuclei were counterstained with DAPI (blue). Right, Means ± SD of two independent experiments (30 cells/each exp) are shown as percentage of MCs double positive for IL-6 and TNF-α mRNA expression (scores from 1 to 3). Data are representative of four mice.

To identify whether MCP4^+^ TAMC subset mainly contributes to cytokine production, RNAscope in situ hybridization technology was combined with immunofluorescence on the same paraffin-embedded colon section obtained from AOM/DSS treated mice. RNA scope was used to reveal mRNA cytokine expression while immunofluorescence to discriminate between mucosal and connective tissue-like MC subsets through the presence of the granule-associated mMCP-1 or mMCP-4, respectively. Focusing on transformed areas of the tissue, we observed that both mucosal mMCP-1^+^ and connective tissue-like mMCP4^+^ MCs express mRNA for IL-6 and TNF-α (Fig. 8). However, quantification of cytokine transcripts showed that more than 50% of mMCP4^+^ MCs and almost 30% of mMCP1^+^ MCs express IL-6 and/or TNF-α transcripts but at different levels (Fig. 8A, scores from 1 to 3), supporting the conclusion that the connective tissue-like MC subset is the main cytokine producer. Of note, images also depicted the expression of both cytokine transcripts (white dots for IL-6 and red dots for TNF-α) in 40% of CTMCs with respect to a 15% of double-positive MMCs (Fig. 8B).

Altogether our findings suggest that the presence of SCF and IL-33 in CRC tissues favor the accumulation of a connective tissue-like MC phenotype which through the production of IL-6 and TNF-α contribute to the establishment of an inflammatory tumor microenvironment.

## Discussion

Compelling evidence support the ability of MCs to switch between different functional subsets in a context-specific and in a localized manner [30]. A mechanism behind this switch could be a result of MC interactions with epithelial cells during an acute inflammatory state or with transformed cells during epithelial tumorigenesis. This means that a pro-fibrotic role of MCs elicited specifically during wound repair can be dysregulated upon transformation: tumors can promote novel pro-inflammatory functions in MCs to reprogram them into a pathogenic state [42, 43].

However, whether the MC phenotype is altered by the TME remains to be elucidated and the exact role of unique MC subset during tumor progression is far from being understood.

Herein, using a murine model of intestinal-type colon cancer we report the presence of high levels of SCF and IL-33 in the TME which coincides with the accumulation of TAMCs with a connective-like phenotype that through the production of TNF-α and IL-6 contributes to a pro-inflammatory environment.

Early findings demonstrate a reduced susceptibility of MC-deficient mice to the development of chemical-induced intestinal tumors [44] and provide evidence for a pro-tumorigenic role of MCs during the development of colitis-associated CRC [45, 46]. However, there are also studies in which MCs appear to play a protective role in CRC tumorigenesis [47–49]. Notably, a recent work from Sakita and colleagues show that MCs could either promote or inhibit the development of colon tumors according to microenvironment stimuli, being tumorigenic in colitis-induced CRC and protective in murine models of sporadic CRC [50]. This finding well fits with the current view that during CRC development MC activity may impact either in a beneficial or harmful fashion depending on the genetic background of mice and the specific tumor models [51].

In our experimental setting, we found that TAMCs release an increased amount of the pro-inflammatory cytokines IL-6 and TNF- α comparing to MC in tumor-free tissue, suggesting a pro-tumorigenic activity (Fig. 1D). This hypothesis is supported by earlier findings showing that intestinal MCs are an important source of TNF-α with which they are involved in a progressive epithelial transformation and polyp development [52–54].

We further showed that inside the colonic lesions the main MC subset is enriched for mMCP4 (Fig. 1E), suggesting a selective accumulation of connective tissue-like MCs. These results are in agreement with previous reports documenting during the transition of polyps to adenocarcinoma a lymphocyte-independent expansion of a subset with characteristic of CTMCs [53, 55, 56].

Moreover, by combining in situ RNA hybridization with immunofluorescence, we firstly demonstrated that mMCP4+ MCs are the main subset responsible for cytokine production in tumor lesions (Fig. 8).

In regard to TME composition, we found high amounts of SCF and IL-33 (Fig. 2A), similar to previous finding obtained in human malignant tumors and a genetic mouse model of CRC [32–36].

The chronic exposure of TAMCs to both soluble factors likely explains the dramatic down-modulation of c-kit and the up-regulation of IL-33R that we have reproducibly observed (Fig. 2B).

SCF and IL-33 act alone or synergistically to elicit survival, differentiation, and cytokine production on both human and mouse MCs [16–19, 57]. Their role is exemplified by the fact that mice lacking c-kit or ST2 have lower numbers of MCs in several tissues [58, 59], while the provision of exogenous SCF or IL-33 increases MC compartment [41, 60]. Accordingly, during intestinal polyposis IL-33 deficiency affects MC accumulation as well as the release of MC-derived proteases and cytokines [36, 59].

Thus, it is likely that in our experimental model the combined action of SCF and IL-33 is responsible for the expansion of a connective-like MC subset inside the tumor either promoting a de novo recruitment and differentiation of immature MC precursors or inducing proliferation of resident mature MC subset(s). Although we have provided in vivo evidence supporting a role for SCF in favoring the accumulation of connective-like MC subset inside the tumor (Fig. 7), future in vivo experiments are required to further underscore the exact role of SCF and IL-33 in MC proliferation and/or MC progenitor recruitment and differentiation during CRC progression.

Regardless, by generating primary cultures from bone marrow- or peritoneal-derived MC precursors we further investigated whether and how SCF and IL-33 regulate MC phenotype and functions.

Bone-marrow-derived precursors differentiated in the presence of SCF show a predominant connective-like phenotype characterized by high transcription levels of mMCP4, 5, and 6 while MCs differentiated in the presence of IL-3 alone showed a significant higher expression of mMCP1 transcripts (Fig. 4 and Fig. S6). However, after differentiation, SCF was unable to further affect protease content (data not shown). Interestingly, stimulation with IL-33 induces a further selective increase of mMCP4, 5 and 6 more evident in BMMCs differentiated with IL-3 and SCF (Fig. 4).

In line with our finding, it has been previously reported that the addition of SCF up-regulates mMCP-4 and -6 on BMMCs obtained with IL-3 alone [61], and that IL-33 increases the expression of the tryptase mMCP-6 in wild type- but not in ST2-deficient bone marrow-derived MCs [62].

During BMMC differentiation, we also observed that in the presence of SCF MCs decrease the expression of c-kit (Fig. 3), mirroring the dramatic c-kit down-modulation that we have observed in vivo on tumor-infiltrating MCs (Fig. 2B). Moreover, they are less prone to degranulate and release cytokines upon FcεRI engagement (Fig. S5), in agreement with previous findings [63, 64]. However, after stimulation with SCF and IL-33 in combination, a superimposable production of IL-6 and TNF-α was observed in all primary MC cultures, regardless the hypofunctional state reported in response to IgE and antigen on IL-3/SCF-derived BMMCs (Fig. 5).

Our findings are consistent with previous data demonstrating a synergistic effect of SCF and IL-33 in MC activation [17] and support the conclusion that during colonic carcinogenesis a sustained stimulation by those soluble factors not only shapes MC phenotype but also promotes the release of IL-6 and TNF-α.

The molecular pathways that regulate the expression of cytokine gene transcripts in MCs are less well defined than those for degranulation. However, it has been previously reported that IL-33 controls the production of IL-6 through ERK1/2 and p38 phosphorylation in a MyD88-dependent manner [39, 40], and that SCF can enhance IL-33-induced production of IL-6 [16, 65].

Accordingly, we found that IL-33 alone was able to induce the phosphorylation of ERK1/2 and p38 together with NF-kB activation (Fig. 6). The activation of these biochemical pathways would explain the ability of IL-33 alone to promote a strong production of IL-6, observed in particular upon stimulation of peritoneal-derived MCs (Fig. 5).

SCF alone in addition to a strong phosphorylation of ERK1/2 observed on both BMMC and PDMC cultures, appears to be able to activate the PI3kinase pathway and to selectively induce STAT3 phosphorylation (Fig. 6).

A plausible interpretation of our findings is that the amplified cytokine production observed by the combined action of SCF and IL-33 depends on the activation of common or unique but convergent signaling pathways.

All together our results underscore the ability of SCF in combination with IL-33 to shape phenotype and function of MCs that through the secretion of IL-6 and TNF-α may contribute to chronic inflammation and disease in the intestinal microenvironment.

In the human gastrointestinal tract two main MC subsets have been categorized on the basis of their protease content: those that contain predominantly tryptase (MCT) mainly present in the lamina propria, and those characterized by the expression of both tryptase and chymase (MCTC) in the submucosa. Of note, in vitro studies show that human MCs are also “tunable” effector cells changing their granule content on the basis of different stimuli including SCF and several cytokines [23].

In CRC patients, no differences in the proportion of these two subsets have been reported so far. However, most studies on the density of tumor-infiltrating MCs have been performed through in situ detection of tryptase-positive cells, without discrimination between MCTC and MCT subsets [66–68]. Thus, no conclusive data exist about the role of a specific MC subset in the carcinogenesis process, emphasizing the need to examine the impact of MCs and their proteases from different perspectives.

In this scenario, an open area of investigation remains if a common progenitor will generate distinct phenotypic and functional MC subsets and/or whether differentiated MCs may exhibit distinct features based on their responsiveness to SCF, IL-33 and/or other specific microenvironment signals.

A deep dissection of MC phenotypic and functional plasticity could offer potential therapeutic targets during CRC progression.

## Materials and methods

Female C57BL/6J mice were purchased from Charles River (Wilmington, MA) and were of 4- to 6-week old by the time of use weighing 20–25 g. All animals were housed in ventilated cages (no more than 5 mice per cage) under specific pathogen‐free conditions and in a controlled environment (12-h daylight cycle, lights off at 18:00) with free access to food and water.

Acute colitis was induced by feeding the mice with 2.5% (wt/vol) dextran sulfate sodium (DSS) (molecular weight 40,000 Da; Merck-Life Sciences, Darmstadt, Germany) in drinking water for 7 days. Monitoring of the percent loss of body weight from day 0 was used to follow the disease course.

To induce colonic tumors, mice were injected by intraperitoneal (i.p.) route with 10 mg/kg Azoxymethane (AOM, Merck-Life Sciences) and, one week later, exposed to 2.5% DSS in the drinking water for 7 days, as previously described [31] but with some modifications. The DSS cycle was repeated 4 times followed by 14 days of fresh water interval (Recovery) (see Fig. S1A). Four cycles of DSS allow the development of intestinal adenomas with a number of colonic tumors ranging from 5 to 12 adenomas.

Thirteen weeks later mice were sacrificed by cervical dislocation after anesthesia.

For blocking experiments, mice were i.p. injected with anti-SCF neutralizing antibody (AB-455-NA R&D Systems) or normal goat control IgG (AB-108-C R&D Systems) (100μg/mouse) three times every 10 days starting one day before the fourth DSS cycle (see Fig. 7A).

All animal studies were conducted in accordance with all relevant ethical regulations for animal testing and research including the Italian code for the care and use of animals for scientific purposes.

The Italian Ministry of Health approved the use of animals for the induction of experimental colitis and colorectal cancer with DSS and AOM/DSS (Authorization n. 698/2021-PR).

### Histological and immunohistochemical analysis

After washing with PBS, the large intestine was cut longitudinally along the main axis, rolled up and immediately fixed in 4% PFA for 24 h. Rolled colons were then extensively washed with PBS and embedded in paraffin. The sections were cut into 5μm thickness, deparaffinized in xylene and hydrated through a graded series of alcohol to water.

Staining with hematoxylin and eosin (Dako Agilent Pathology solutions, Santa Clara, CA) was performed under standard conditions. Dehydration steps were performed with increasing concentrations of ethanol. Sections were mounted with Entellan anhydrous mounting medium (Merck Life Sciences) and images were acquired using a Leica DM1000 microscope.

For immunohistochemistry (IHC), antigen retrieval was performed by heating slides in citrate buffer for 20 min and then cooling to room temperature. Endogenous peroxidase activity was quenched with hydrogen peroxide (3% in Methanol) for 10 min at room temperature. After blocking with 1% FCS, tissue slides were incubated with the anti-Ki-67 antibody (Thermo Scientific, Waltham, MA) diluted 1:250 in PBS overnight at 4 °C. Detection was carried out with Mouse-to-Mouse HRP (DAB) staining system (ScyTek Laboratories, Logan, UT), as previously described [69]. Negative controls were processed in the same manner but without primary antibody.

For toluidine blue staining, slides were dewaxed and rehydrated and stained with 0.5% w/v toluidine blue (Merck Life Sciences) in 1% Sodium chloride for 2–3 min and washed with distilled water. Slides were dehydrated and mounted in Entellan mounting medium.

### Immunofluorescence and RNAscope

Paraffin-embedded sections were dewaxed, rehydrated and antigen-retrieval was performed in citrate buffer for 20 min at 100 °C. Sections were incubated overnight at 4 °C with rat anti-mouse MCP1 Ab (E-bioscience) diluted in 5% normal goat serum or with goat anti-mouse MCP4 Ab (Abcam, Cambridge, United Kingdom) diluted in 1% normal donkey serum after blocking with 10% of normal goat serum or normal donkey serum for 1 h, respectively. AlexaFluor 488-conjugated goat anti-rat or AlexaFluor 488-conjugated donkey anti-goat (Thermo Scientific) Ab was used to reveal mMCP1 and mMCP4, respectively. Where indicated, AlexaFluor 647-conjugated donkey anti-goat Ab was used to reveal mMCP4. After counterstaining with DAPI (Thermo Scientific) for 15 min, sections were mounted with Prolong Diamond antifade mounting solution (Thermo Scientific). Imaging was carried out with an inverted Olympus IX73 microscope equipped with a X-Light V3 spinning disk (Crestoptics, Rome, Italy) using a Prime BSI Express Scientific CMOS camera. Images of the entire tissue sections were collected as 2048 × 2048 pixel files by using a UPLANFL 20×/0.45NA objective (Olympus, Tokyo, Japan) and MetaMorph V7.8.0 software (Molecular Devices, San Josè, CA) and then processed with Fiji ImageJ software. The number of MCs was automatically analyzed in each image using Fiji software. Where indicated, images were acquired with Zeiss LSM980 confocal microscope using a 20×/0.8NA objective (Zeiss, Jena, Germany).

RNAscope was performed using RNAscope Multiplex Fluorescent V2 Assay **(**ACDBio, Minneapolis, MN) combined with immunofluorescence according to the manufacturer’s instructions.

Briefly, sections were deparaffinized and hydrated followed by Hydrogen Peroxide treatment for 10 min at room temperature. To improve RNA exposure tissues were immersed in 1X antigen retrieval 20 min 100 °C in the steamer and transferred to 100% ethanol for 3 min. Slides were dried at 40 °C for 5 min. Protease plus solution was incubated for 30 min at 40 °C before RNA probes (Mm-TNFα, Cat No. 311081 and Mm-Il6-C2, Cat No. 315891-C2) incubation for 2 h at 40 °C. Amplification steps were performed following the manufacturer’s instructions and TNF-α mRNA signal was revealed by HRP-C1 incubation 15 min at 40 °C followed by Opal690 fluorophore 30 min at 40 °C while IL-6 signal was revealed by HRP-C2 followed by Opal570 fluorophore. All incubations were performed in a pre-warmed HybEZHumidity Control Tray. After blocking unspecific binding, mMCP1 and mMCP4 immunofluorescent staining was performed on the same sections, as previously described. Slides were then counterstained with DAPI and mounted with Prolong Diamond mounting medium.

Confocal images were acquired with a Zeiss LSM980 confocal microscope using a 63×/1.35NA oil-immersion objective (Zeiss, Jena, Germany). The pinhole was set at one Airy unit and 3 sequential scans were created to avoid overlapping spectra. Z-stack acquisition (0.2 μm step) was performed based on mMCP1/MCP4 signal at 4 μs/pixel with 2 frames averaging and bidirectional scanning.

All quantified images were acquired by using identical confocal settings. Projected images were generated from unprocessed z-stacks and puncta count was performed using Fiji ImageJ software (NIH). Regions of interest (ROI) were created based on mMCP1/MCP4 signals and the number of puncta in each ROI was automatically analyzed. The same threshold was used in all images.

Cytokine-producing cells were identified according to a scoring system described in the ACDBio protocol, with some modifications. Briefly, the four-grade scoring system was defined as: score 0, no staining or 1 dot/cell; score 1, 2-4 dots/cell; score 2, 4-10 dots/cell; score 3, more than 10 dots/cell. Cells with score 0 were considered negative for cytokine expression. Score from 1 to 3 is a measure of increased cytokine production. Cells were considered double positive for IL-6 and TNF-α expression if at least two dots of each cytokine/cell were revealed.

### Isolation of colon infiltrating immune cells and flow cytometric analysis

To analyze immune infiltrate in the gut, colons were dissected from mice, cut longitudinally upon flushing the luminal contents and incubated in 5 mM EDTA and 10 mM Hepes at 37 °C for 30 min to remove the epithelial layer.

Lamina propria leukocytes were isolated by mechanical dissociation and enzymatic digestion in RPMI medium containing 0.5 mg/ml DNase I and 0.25 mg/ml Liberase TL (Merck Life Sciences) for 30 min at 37 °C and strained (100-µm filter). Leukocytes were enriched using 40% Percoll (Merck Life Sciences). Tumor-infiltrating leukocyte were isolated with the same protocol except for EDTA/Hepes step.

The following Abs were used for flow cytometric analysis: anti-CD3-FITC; anti-CD19-FITC; anti-NK1.1-FITC; anti-cKit-APC (clone 2B8); anti-cKit-PE (clone 2B8); anti-FcεRIα-PE-Cy7, anti-FcεRIα-Pacific blue (all from Biolegend, Waltham, MA); anti-CD11b-FITC and anti-CD45-PerCP-Cy5.5 (from Thermo Scientific); anti-IL-33R PE (BD Biosciences, Drive Franklin Lakes, NJ).

Dead cells were excluded using APC-H7-conjiugated Fixable viability Dye (Thermo Scientific).

In experiments of cytokine production, isolated cells were treated with Cell Stimulation Cocktail (ThermoFisher), containing PMA/Ionomycin for 2 h at 37 °C, stained for cell surface markers and then fixed and permeabilized using the Cytofix/cytoperm kit (BD Biosciences) before intracellular staining with anti-TNF-α-PE-Cy7 and anti-IL-6-APC.

Samples were acquired by FacsCantoII and Flow cytometric analysis was performed with FlowJo 10 software (BD Bioscience).

### ELISA assays

Colon was excised from each mouse, and a 1 cm of tissue was homogenized in 500 μl PBS containing protease inhibitors (Thermo Scientific) and Triton X-100 at the final concentration of 1%. Samples were frozen at −80 °C for 1 h, thawed and centrifuged at 13,000 rpm 15 min 4 °C to remove particulates. Protein concentration was assessed by Bradford assay. Elisa kits (Quantikine ELISA kits, R&D Systems, Minneapolis, MN) were used to quantify IL-33 and SCF according to the manufacturer’s instructions.

### Murine mast cell cultures

Mouse BMMCs were differentiated and cultured as previously described with some modifications [37, 38]. Bone marrow cells were isolated from femurs of C57BL/6 mice, differentiated in complete RPMI, and supplemented with 10% Fetal Calf Serum (FCS), 100 U/mL penicillin/streptomycin, 1% glutamine and with 30 ng/mL recombinant murine IL-3 (Peprotech, London, United Kingdom) alone or together with 25 ng/mL recombinant murine SCF (Peprotech) at 37 °C under 5% CO_2_. Cultures were passaged every 3 days by resuspending the pelleted cells in fresh culture medium at a concentration of 1 × 10^6^/ml. After 4-6 weeks of culture, BMMC purity was evaluated by flow cytometry as the percentage of FcεRI and cKit double-positive cells using anti-cKit-APC-H7 (BD Biosciences) and anti-FcεRIα-Pacific blue (Biolegend) Abs. BMMCs were used at a purity of more than 90%.

To differentiate MCs from peritoneal precursors, peritoneal cavity lavage with 5 ml of cold PBS was performed to euthanized WT mice. Cells were centrifuged and washed, then cultured 1 × 10^6^/ml cells in complete RPMI medium, mouse recombinant IL-3 (30 ng/ml, Peprotech) and SCF (25 ng/ml, Peprotech). Twenty-four hours later, nonadherent cells were removed and fresh culture medium was added to adherent cells. Three days later, nonadherent cells were harvested, pelleted, and resuspended in a fresh culture medium. The same procedure was repeated twice a week for two weeks. MC purity was evaluated by flow cytometry and cultures more than 90% double positive for FcεRI and c-Kit were used for in vitro experiments.

### Primary culture cell stimulation and cytokine detection flow cytometric analysis

For intracellular cytokine detection, primary MC cultures were starved overnight in RPMI complete medium supplemented with 15 ng/ml IL-3 and then treated with 100 ng/ml of IL-33 and/or SCF alone or in combination in the presence of Brefeldin A 5 μg/ml (Merck Life Sciences) for 6 h at 37 °C.

Mouse mast cells were harvested, fixed and permeabilized using Cytofix/Cytoperm kit (BD Biosciences) and then stained with anti-TNFα-PECy7 and IL-6-APC Abs.

### Western blot analysis

MCs were starved overnight in RPMI 1640 supplemented with 5%FCS and 15 ng/ml recombinant murine IL-3 and then stimulated with IL-33 (100 ng/ml) and/or SCF (100 ng/ml) alone or in combination for 15 min at 37 °C. Cells were homogenized in fresh prepared RIPA lysis buffer (Tris-HCl 50 mM, NaCl 150 mM, Triton X-100 1%, SDS 0.1%, DOC 0.5%, EDTA 1 mM, EGTA 1 mM, MgCl2 5 mM, leupeptin 5μg/ml, PMSF 1 mM, aprotinin 5μg/ml, NaF 5 mM, Na_3_VO_4_ 1 mM). Lysates were centrifugated at 13,000 g for 20 min to eliminate debris, resolved by SDS-polyacrylamide gel (PAGE) and proteins were electro-blotted, as previously described [70].

Immunoreactive bands were visualized on the nitrocellulose membranes, using horseradish-peroxidase-linked/coupled donkey anti-rabbit or sheep anti-mouse IgG (GE Healthcare, Chicago, IL) and the Clarity max, western ECL substrate (Biorad, Hercules, CA) based on manufacturer’s instructions and exposed to Invitrogen iBrightTM Imaging Systems (Thermo Scientific).

The following Abs were used: anti-pSTAT3, anti-STAT3, anti-pAkt S473/D9E, anti-Akt, anti-pp38 T880/Y182, anti-p38, anti-pp44/42 (ERK1/2) (T202/Y204) and anti-P44/42 (ERK1/2) (all from Cell Signaling, Danvers, MA); anti-Ikb-α (Santa Cruz Biotechnologies, Dallas, TX); anti-actin (Merck Life Sciences). Densitometric analysis was performed using FIJI Image J software.

### Degranulation assay

Degranulation was determined by measuring the release of β-hexosaminidase, as previously described [38].

Briefly, BMMCs were sensitized with 1 μg/mL IgE for 1 h at 37 °C in complete media. Cells were washed twice with Tyrode’s buffer (10 mM Hepes buffer pH 7.4, 130 mM NaCl, 5 mM KCl, 1.4 mM CaCl_2_, 1 mM MgCl_2_, 5.6 mM glucose and 0.1% bovine serum albumin) and plated in 96-well plates at 3 × 10^6^/mL in 100 μL of Tyrode’s buffer. Cells were then treated with DNP-HSA (100 and 1000 ng/mL) for 30 min at 37 °C.

The enzymatic activity of β-hexosaminidase in supernatants and cell pellets was measured with p-nitrophenyl N-acetyl-D-glucosaminide (NAG) in 0.1 M sodium citrate for 30 min at 37 °C. The release of the product 4-p-nitrophenol was detected by absorbance at 405 nm.

### Quantitative reverse transcriptase polymerase chain reaction

MCs were stimulated for 48 h at 37 °C with 3 ng/ml and 10 ng/ml IL33 and total RNA was extracted using Total RNA mini kit (Geneaid, New Tapei City, Taiwan). Total RNA (500ug) was used for cDNA synthesis using Moloney murine leukemia virus reverse transcriptase (Promega, Madison, WI) and real-time PCR was performed using the ABI Prism 7900 Sequence Detection system (Applied Biosystems, Waltham, MA) with the following primers: mMCP1 (Mm04213434_g1); mMCP4 (Mm07306493_g1); mMCP5 (Mm00487638_m1); mMCP6 (Mm01301240_g1). The level of gene expression was measured by subtracting the threshold cycle (Ct) of the gene of interest from the Ct of GAPDH that served as housekeeping gene. Ct of the untreated sample was used as the calibrator and the fold change was calculated, as previously described [71].

### Statistical analysis

Student’s *t* test and one-way ANOVA followed by Tukey’s multiple-comparisons tests were used to quantify statistical deviation between experimental groups, as indicated in figure legends. Prism 7 software (GraphPad Software, San Diego, CA) was used. Graphs show mean values, and all error bars represent the SD.

Asterisks denote significant differences. *p < 0.05; **p < 0.01; ***p < 0.001; ****p < 0.0001.

### Supplementary information

Supplementary Figures

aj-checklist

## Data Availability

All data generated or analyzed during this study are included in this published article and its supplementary information files.
