# Supplementary material for: SCF and IL-33 regulate mouse mast cell phenotypic and functional plasticity supporting a pro-inflammatory microenvironment
Source: Cell Death Dis. 2023 Sep 20;14(9):616. doi: 10.1038/s41419-023-06139-7 (PMC10511458; doi:10.1038/s41419-023-06139-7)
Supplement: Supplementary file 1 — Supplementary Figures [file 41419_2023_6139_MOESM1_ESM.pdf]

SCF and IL-33 regulate mouse mast cell phenotypic and functional plasticity supporting a pro-inflammatory microenvironment

Rosa Molfetta[1*], Mario Lecce[1&§], Nadia D. Milito[1§], Erisa Putro[1], Giuseppe Pietropaolo[1], Caterina Marangio[1], Gianluca Scarno[$1], Marta Moretti[2], Enrico De Smaele[2], Tiziana Santini[3], Giovanni Bernardini[1], Giuseppe Sciumè[1], Angela Santoni[1,4], and Rossella Paolini[1*]

[1]Department of Molecular Medicine, Sapienza University of Rome, Laboratory affiliated to Istituto Pasteur Italia - Fondazione Cenci Bolognetti, Rome, Italy.
[2]Department of Experimental Medicine, Sapienza University of Rome, Viale Regina Elena 324, 00161, Rome, Italy.
[3]Department of Biology and Biotechnologies "Charles Darwin", Sapienza University of Rome, Rome, Italy.
Center for Life Nano- & Neuro-Science, Fondazione Istituto Italiano di Tecnologia, Rome, Italy.
[4] IRCCS Neuromed, Pozzilli, 86077 Isernia, Italy.

[&]Current address: Leibniz Institute for Immunotherapy-Division of functional immune cell modulation, Franz-Josef-Strausse, D-93053 Regensburg.
[$]Current address: Zuckerman Research Center, 417 East 68th St., 10065 New York.

[§]These authors equally contributed to the work: Mario Lecce, Nadia Domenica Milito.

*Correspondence to Rossella Paolini or Rosa Molfetta - Department of Molecular Medicine, University of Rome "La Sapienza" - Viale Regina Elena 291, 00161 - Rome, Italy.
Telephone: +39-0649255670 FAX: +39-0644340632
Email: rossella.paolini@uniroma1.it or rosa.molfetta@uniroma1.it

Supplementary Figures S1 to S6

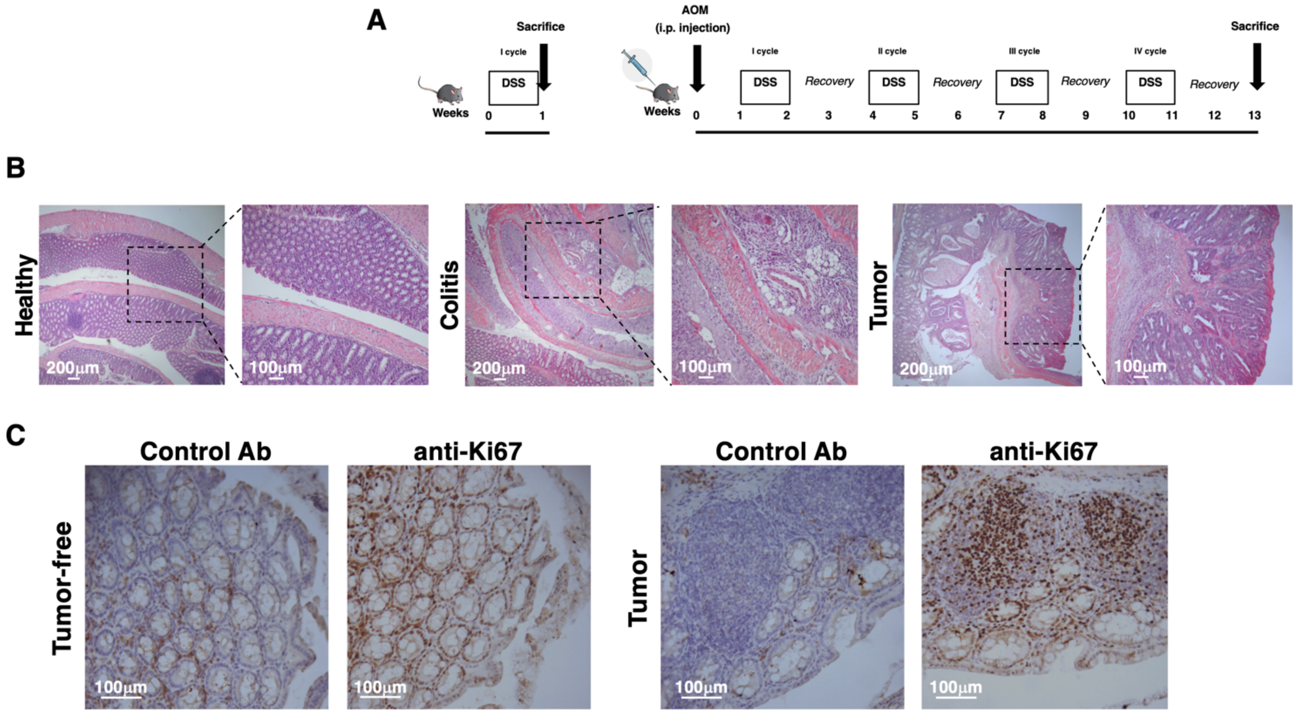

Figure S1. Schematic representation of DSS and AOM/DSS treatment and morphological analysis of murine colon sections.

(A) To induce acute colitis DSS was administered in drinking water for one week and mice were sacrificed and analysed immediately (left). To induce colonic adenomas one week after the intraperitoneal injection of AOM, DSS was administered in drinking water for one week followed by two weeks of mice recovery from inflammation. These cycles were repeated for 4 times and mice were sacrificed at the end of the last recovery period.

(B) Representative images of hematoxylin and eosin staining of the colon sections of untreated mice (Healthy), DSS-treated mice (Colitis) and AOM/DSS treated mice (Tumor) acquired with 4x (left panels) and 10x (right panels) objectives.

(C) Consecutive tissue sections from AOM/DSS treated mice were stained with anti-Ki67 Ab or control Ab, as indicated, counterstained with hematoxylin and acquired with a 20x objective.

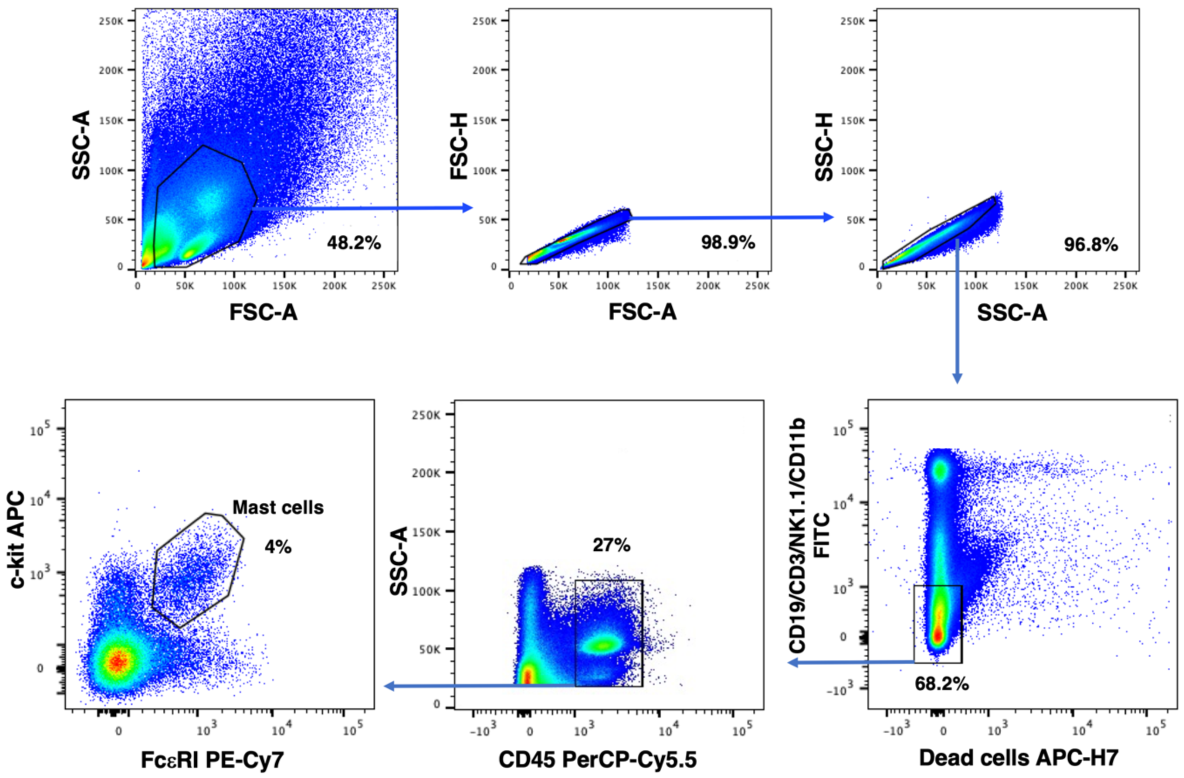

Figure S2. Flow cytometric gating strategy to identify MCs out of CD45+ cells isolated from tumoral colon tissue.

Cells isolated from tumoral and non-lesional colon tissues were first gated based on morphologic characteristics (upper panels) and then the surface expression of c-kit (anti-cKit-APC, clone 2B8) and FcεRI (anti-FcεRI α chain-PECy7) was analyzed after excluding CD3+/CD19+/CD11b+/NK1.1+ (all FITC-conjugated Abs) and dead cells (APC-H7-conjugated Fixable viability Dye) and finally positive gating for CD45+ cells (anti-CD45-PerCP-Cy5.5).

Where indicated, anti-cKit-PE and anti-FcεRIα-Pacific blue were used to analyse MCs.

A

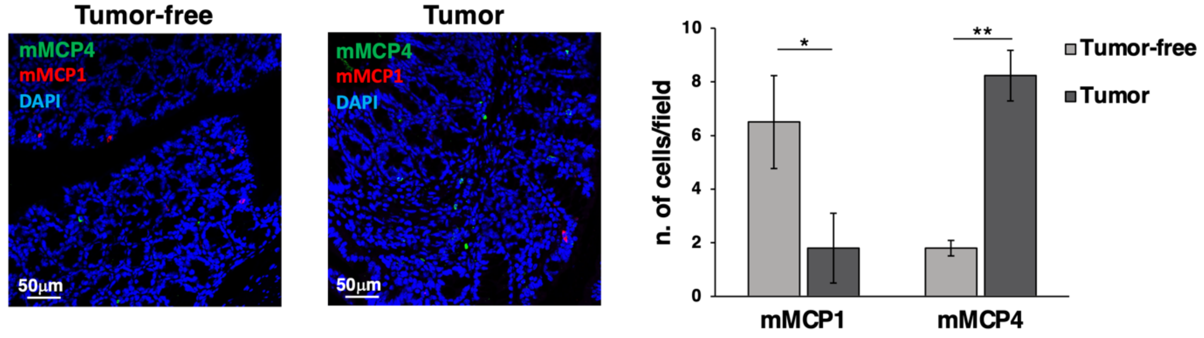

B

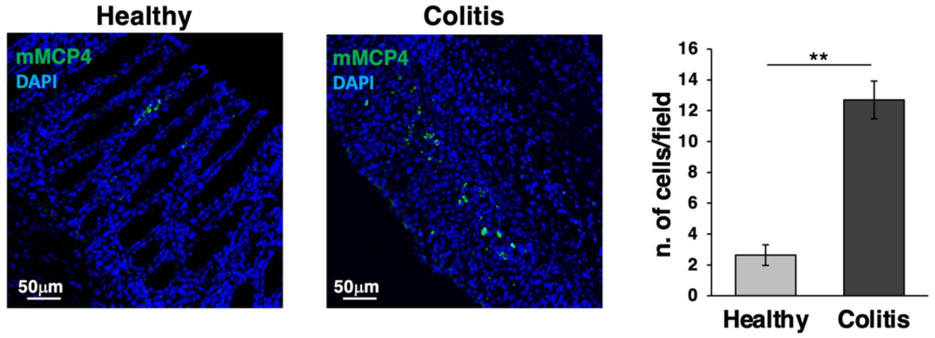

Figure S3. MC subset localization in AOM/DSS and DSS mouse model.
A: Colon paraffin-embedded sections from AOM/DSS treated mice were stained with anti-mouse MCP4 Ab followed by Alexa Fluor 488 secondary Abs (green) and anti-mouse MCP1 Ab followed by Alexa Fluor 647 secondary Abs (red). Nuclei were counterstained with DAPI (blue) and acquired with Zeiss LSM980 microscope using a 20x objective. The frequencies of MCs positive for mMCP4 and MCP1 proteases were analysed in 20 fields randomly acquired from tumor-free tissue and tumor lesions and shown as mean +/-SD cells/field. Paired Student' t test *p<0.05; **p. <0.01.
Data are representative of 2 independent experiments with at least 3 mice/group.
B: Colon paraffin-embedded sections from DSS treated mice were stained with anti-mouse MCP4 Ab followed by Alexa Fluor 488 secondary Abs. Nuclei were counterstained with DAPI and acquired with Olympus IX73 microscope equipped with a X-Light V3 spinning disk using a 20x objective. Data quantification in healthy tissue and colitis is shown on the right as mean+/- SD of 20 randomly acquired fields from 2 independent experiments with at least 3 mice/group.
Paired Student' t test **p<0.01.

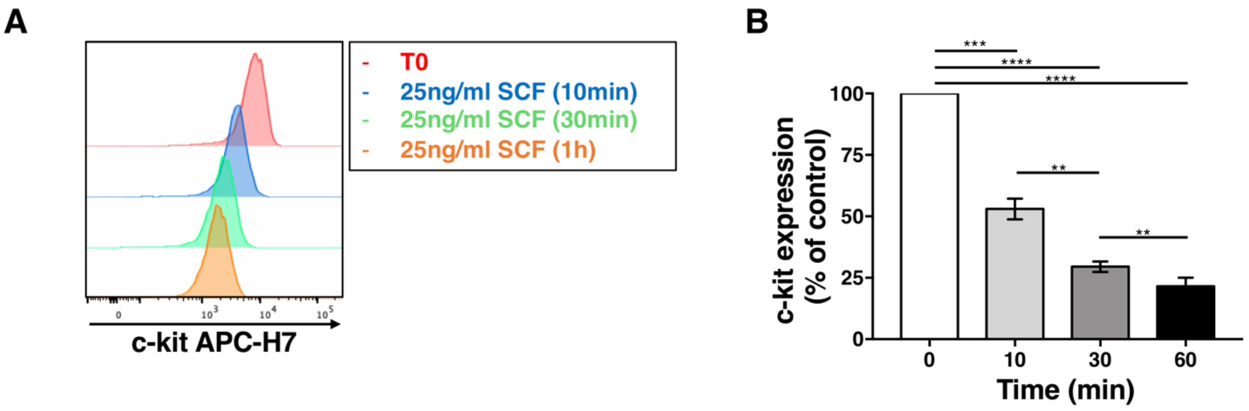

Figure S4. BMMCs differentiated in IL-3 alone downmodulate c-kit after SCF stimulation. BMMCs were generated upon culture of BM-derived MC precursors in the presence of IL-3 alone (30ng/ml) for 4 weeks. The cells were then challenged with SCF (25ng/ml) for the indicated length of times and c-kit surface expression was evaluated by flow-cytometry. Representative histograms (A) and the means +/- SD of three independent experiments (B) are shown.

One-way ANOVA $^{**}p<0.01$; $^{***}p<0.001$; $^{****}p<0.0001$.

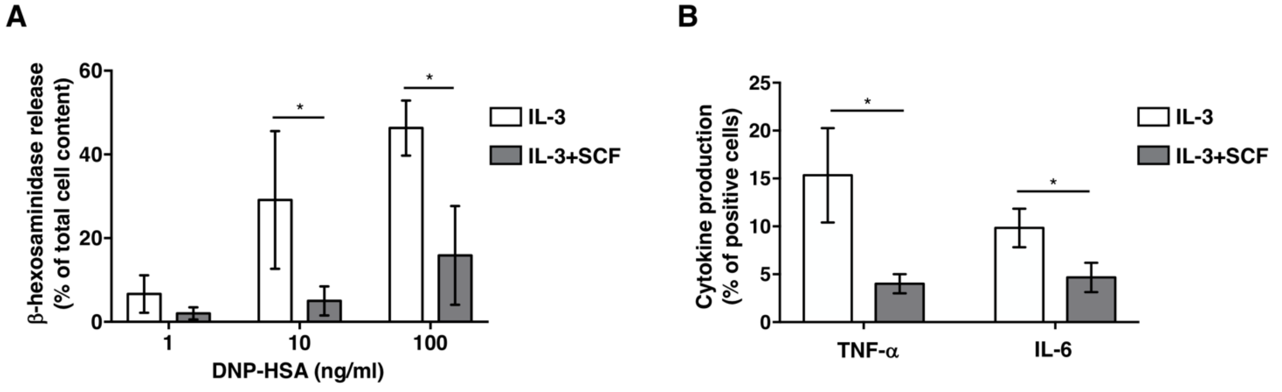

Figure S5. Prolonged exposure of developing BMMCs to SCF induces a hypo-responsive phenotype upon IgE/antigen stimulation.

BMMCs were cultured in IL-3 (30ng/ml) alone or in the presence of SCF (25ng/ml), as indicated, for 4 weeks. MCs were then starved of cytokines overnight and concurrently sensitized with IgE specific for DNP.

(A) Cells were challenged with the indicated concentrations of multivalent antigen (DNP-HSA) for 30 min and degranulation was evaluated as net β-hexosaminidase release.

(B) The IgE-sensitized cells were challenged for 6h with 100 ng/ml DNP-HSA in the presence of Brefeldin A and the cytokine expression was evaluated by flow cytometry upon permeabilization. The data represent means +/- SD of three independent experiments. Paired Student' t test *$p<0.05$.

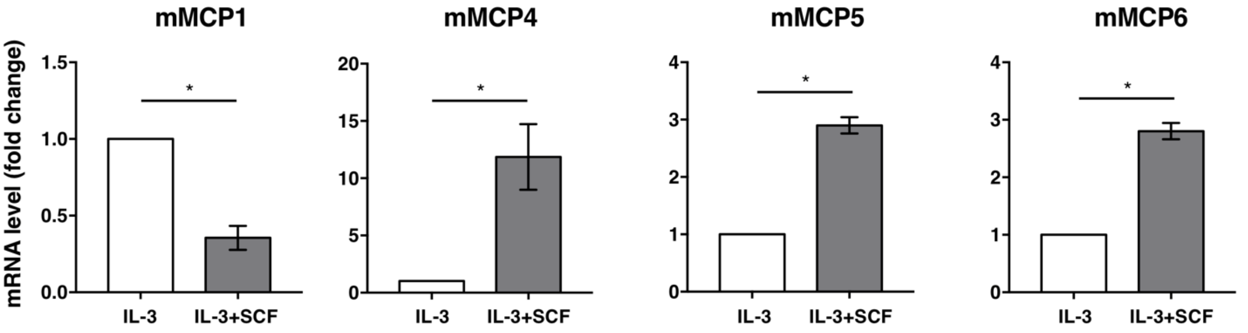

Figure S6. Basal expression of protease transcripts along BMMC cultures.
The expression of mMCP1, mMCP4, mMCP5 and mMCP6 mRNA transcripts was evaluated by real-time PCR and compared in BMMCs cultured in IL-3 (30ng/ml) alone or in the presence of SCF (25ng/ml) for 4 weeks. mRNA expression levels in BMMC cultured in the presence of IL-3 alone were arbitrary set to 1. The data represent means +/- SD of three independent experiments. Paired Student' t test $*p<0.05$.
